# People adaptively use information to improve their internal states and external outcomes

**DOI:** 10.1016/j.cognition.2022.105224

**Published:** 2022-11

**Authors:** I. Cogliati Dezza, C. Maher, T. Sharot

**Affiliations:** a Department of Experimental Psychology, Faculty of Brain Sciences, University College London, 26 Bedford Way, London WC1H 0AP, UK; b Department of Experimental Psychology, Ghent University, Henri Dunantlaan 2, Ghent, BE, Belgium; c The Max Planck UCL Centre for Computational Psychiatry and Ageing Research, University College London, 10-12 Russell Square, London, WC1B 5EH, UK; d Department of Brain and Cognitive Sciences, Massachusetts Institute of Technology, 43 Vassar St, Cambridge, MA 02139, USA

**Keywords:** Information-seeking, Affect, Uncertainty, Agency

## Abstract

Information can strongly impact people's affect, their level of uncertainty and their decisions. It is assumed that people seek information with the goal of improving all three. But are they successful at achieving this goal? Answering this question is important for assessing the impact of self-driven information consumption on people's well-being. Here, over five experiments (total *N* = 727) we show that participants accurately predict the impact of information on their internal states (e.g., affect and cognition) and external outcomes (e.g., material rewards), and use these predictions to guide information-seeking choices. A model incorporating participants' subjective expectations regarding the impact of information on their affective, cognitive, and material outcomes accounted for information-seeking choices better than a model that included only objective proxies of those measures. This model also accounted for individual differences in information-seeking choices. By balancing considerations of the impact of information on affective, cognitive and material outcomes when seeking knowledge, participants became happier, more certain and made better decisions when they sought information relative to when they did not, suggesting that the actual consequences of receiving information aligned with their subjective expectations.

## 1 Introduction

A vast amount of information is currently available to people. This includes personalized information, such as information about one's genetic makeup or financial prospects. People thus need to make frequent decisions about which information they would like to receive and which they would rather avoid. Consequently, there is a pressing need to investigate how these choices influence how people feel, think, and act.

In particular, the decision to seek or avoid information can have significant impact on people's internal states (e.g., affect and cognition) and external (e.g., material reward) outcomes. As for internal states, information can induce emotions such as joy (e.g., consider the impact of reading positive reviews of your work) or fear (e.g., hearing about a dangerous mutations of the coronavirus). Information can also increase people's confidence in their ability to comprehend the world around them, or cause confusion (e.g., reading conflicting reports about the efficacy of a vaccine). As for external outcomes, people can use information to select actions that will lead to extrinsic rewards and avoid losses (e.g., people can use information to make profitable financial investments).

A growing literature has begun to characterize how people make information-seeking decisions. For example, it has been shown that people want information more when it will likely reveal good rather than bad news (Charpentier, Bromberg-Martin, & Sharot, 2018; Karlsson, Loewenstein, & Seppi, 2009; van Lieshout, de Lange, & Cools, 2020; van Lieshout, Traast, de Lange, & Cools, 2021), when uncertainty is high (Bromberg-Martin & Monosov, 2020; Chater & Loewenstein, 2016; Cogliati Dezza, Yu, Cleeremans, & Alexander, 2017; Crupi, Nelson, Meder, Cevolani, & Tentori, 2018; Friston, 2010; Golman, Gurney, & Loewenstein, 2020; Golman & Loewenstein, 2018; Gottlieb, Oudeyer, Lopes, & Baranes, 2013; Kidd & Hayden, 2015; Kobayashi, Ravaioli, Baranes, Woodford, & Gottlieb, 2019; Oudeyer, Lopes, Kidd, & Gottlieb, 2016; Schulz et al., 2019; Schwartenbeck et al., 2019; van Lieshout, de Lange, & Cools, 2021), and when the instrumental utility of information (e.g., the ability of information to guide future actions towards high rewards) is great (Cogliati Dezza, Cleeremans, & Alexander, 2022; Kobayashi & Hsu, 2019; Stigler, 1961; Wilson, Geana, White, Ludvig, & Cohen, 2014). Presumably this is because people are motivated to use information to positively impact their affect, reduce subjective sense of uncertainty and make decisions that lead to greater rewards (Bromberg-Martin & Sharot, 2020; Sharot & Sunstein, 2020).

Yet, we know surprisingly little about the *consequences* of those decisions. Do people succeed in improving their internal states and external outcomes through information-seeking? We know that focusing on the consequences of information decisions can reduce people's curiosity towards aversive uncertain events (Hsee & Ruan, 2016). But do they accurately estimate the downstream impact of information on their internal states and external outcomes? To answer these questions, it is necessary to measure people's *expectations* of how information will impact them, record their information-seeking *choices* and the *subjective consequences* of those choices. This has yet to be done. In fact, attempts to predict people's information-seeking choices have relied almost exclusively on objective measures. For example, we (Charpentier et al., 2018; Cogliati Dezza et al., 2017; Cogliati Dezza, Noel, Cleeremans, & Yu, 2021) and others (Bromberg-Martin & Hikosaka, 2009; Bromberg-Martin & Hikosaka, 2011; Gershman, 2019; Iigaya, Story, Kurth-Nelson, Dolan, & Dayan, 2016; Kobayashi et al., 2019; Kobayashi & Hsu, 2019; Wu, Schulz, Speekenbrink, Nelson, & Meder, 2018) have mathematically quantified the likelihood that information will reveal a desirable outcome, the amount of uncertainty it can resolve, and its instrumental utility, and used those measures to predict information-seeking behavior. The rationale behind this approach is that these mathematical quantities likely reflect people's *subjective* evaluations. However, it is unknown how good these proxies really are. Indeed, subjective expectations about the consequences of an event do not always align with the objective estimates of those consequences (e.g., impact bias, Wilson & Gilbert, 2005). Moreover, if people's *subjective* expectations regarding the impact of information is driving their decisions, a model that use those subjective estimates should predict information-seeking choices better than their objective proxies.

Here, we examine whether people anticipate the impact of information on their internal states and external outcomes and whether those subjective estimates are better predictors of information-seeking choices than objective measures. We further test whether people's information-seeking choices improve their affect, reduce their subjective uncertainty, and improve their decisions. To do so we develop a task that allows us to measure and manipulate these factors, such that the correlations among these variables were minimized. Because different people will have different expectations about how information will impact them and different reactions to information, to fully explain information-seeking we must measure these subjective reactions and examine how they drive choices.

## 2 Methods and material

### 2.1 Participants

#### 2.1.1 Experiment 1 - main study

60 participants (mean age = 25.6, SD = 8, 22 females) completed the study on the online platform Prolific Academic (https://www.prolific.co/). Six participants purchased information on fewer than 10 trials and were excluded from the analysis due to lack of variance in the predicted variable, which made it difficult to run comparison tests and calculate reliable estimates. The final sample was composed of 54 participants (mean age = 24.8, SD = 7.6, 16 females). All participants were paid £7.50 per hour for their participation. The study was approved by the departmental ethics committee at UCL.

#### 2.1.2 Experiment 2 - replication

150 participants (mean age = 26.9, SD = 8.4, 71 females) were recruited on Prolific Academic. N was determined based on a power analysis of data from Experiment 1. Estimated 120 subjects for an alpha = 0.05, power = 0.95 with 20% added to account for potential unusable data and rounded up. Thirteen participants purchased information on fewer than 10 trials and were excluded from the analysis due to lack of variance in the predicted variable. One participant pressed the same button for every subjective affect rating and was excluded from the analysis for the aforementioned reason. The final sample was composed of 136 participants (mean age = 27.0, SD =8.5, 60 female). All participants were paid £7.50 per hour for their participation. The study was approved by the departmental ethics committee at UCL.

#### 2.1.3 Experiment 3,4 - control study I & II

In *Experiment 3*, 124 participants (mean age = 43.2, SD = 13.6, 69 females), and in *Experiment 4*, 204 participants (mean age = 26.3, SD = 9.2, 74 females) were recruited on Prolific Academic. Five subjects in *Experiment 3* and 4 subjects in *Experiment 4* purchased information on fewer than 10 trials and were excluded from the analysis. The final sample was composed of 119 (mean age = 43, SD = 13.4, 66 females) and 200 participants (mean age = 26.4, SD = 9.2, 72 females), respectively. All participants were paid £7.50 per hour for their participation. The study was approved by the departmental ethics committee at UCL.

#### 2.1.4 Experiment 5 – control Study III

189 participants (mean age = 38.7, SD = 12.4, 112 females) were recruited on Prolific Academic. N was determined based on a pilot study. Thirteen participants purchased information on fewer than 10 trials and were excluded from the analysis. The final sample was composed of 176 participants (mean age = 38.8, SD =12.5, 104 female). All participants were paid £7.50 per hour for their participation. The study was approved by the departmental ethics committee at UCL.

### 2.2 Behavioral task

**Fig. 1:**
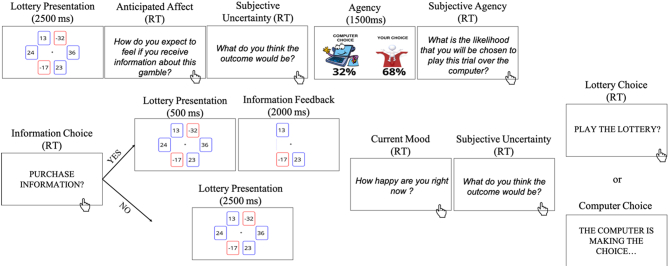
Information-seeking task. A lottery with 6 cards was displayed for 2500 ms. Each card displayed a possible gain or loss. While the lottery was still on screen, participants were asked to rate their expected mood if they end up purchasing information and how likely they believed they were to gain or lose. The latter rating was used to compute subjective uncertainty of the outcomes. Next, while the lottery was still on screen, the objective likelihood that participants would have the opportunity to play or pass on the lottery on a given trial (i.e., objective agency) was presented for 1500 ms, followed by a subjective rating of the likelihood participants thought they had of making that decision. Next, while the lottery was still on screen, participants indicated whether they wanted to purchase information on a 6-point Likert scale (−3 – “definitely no”, −2 – “no”, −1 – “somewhat no”, 1 – “somewhat yes”, 2 – “yes”, 3 – “definitely yes”). If participants selected any of the “YES” options, the six cards were shown again for 500 ms and then 3 cards were removed. If participants selected any of the “NO” options, then all six cards were displayed for 2500 ms. Participants were then asked to rate their current mood and how likely they believed the outcome would be either a gain or loss. The latter rating was used to compute subjective uncertainty of the outcomes. Then, participants were either given the option to decide whether to play the lottery or pass, or the computer did so for them.

Participants were told to imagine they were playing a lottery game in a casino and they could improve their bonus payment by winning points on the game. Participants started the game with 0 points. The lottery game consisted of 90 trials. On each trial, a lottery composed of 6 cards was shown on screen for 2500 ms (Fig. 1). Cards were either blue or red. The blue cards indicated possible gain and the red cards indicated possible loss. Each card had a number on it, indicating the magnitude of associated loss or gain. Participants were told that one of the cards had a star on the back of it (i.e., the outcome card), which they could not see. If the lottery was played out the number on that card would determine the outcome on that trial. Each participant saw the same set of lotteries, the order of which was random and differed across participants.

After the lottery was displayed on the screen, participants were asked to rate their anticipated affect by indicating “How do you expect to feel if you receive information about this gamble?” on a 7-point scale (i.e., 0 – “very unhappy”, 1 – “unhappy”, 2 – “somewhat happy”, 3 – ‘neutral”, 4 – “somewhat happy”, 5 – “happy”, 6 – “very happy”) and to indicate “What do you think the outcome would be?” on a 7-point (i.e., 0 – “certainly loss”, 1 – “loss”, 2 – “likely loss”, 3 – “not sure”, 4 – “likely gain”, 5 – “gain”, 6 – “certainly gain”). The latter query was used to compute subjective uncertainty (**Analysis**). Next, they were shown the probability that they would be able to make that decision (i.e., objective agency; Fig. 1A). Participants were told that on some trials they could decide to play the lottery or pass and on some other trials the computer would randomly decide for them. We introduced the choice likelihood manipulation to modulate the usefulness of information – that is, the extent to which information can impact future choices (what we call instrumental utility). If, for example, the probability that the subject will be the one to make a lottery choice is 0%, purchasing information cannot be useful to improve future choices as the participant has not control over the outcome. They were then asked to rate their subjective expectation of this probability by indicating “What is the likelihood that you will be chosen to play this trial over the computer?” on a 7 – point scale: 0 – “very unlikely”, 1 – “unlikely”, 2 – “somewhat unlikely”, 3 – “neither likely nor unlikely”, 4 – “somewhat likely”, 5 – “likely”, 6 – “very likely”. This later measure allows us to compute participants’ interpretation of the likelihood, which can differ across individuals.

Prior to the lottery choice, participants were asked to indicate whether they wanted to purchase information about the upcoming lottery choice on a 6-point Likert scale: −3 (“definitely no”), −2 (“no”), −1 (“somewhat no”), 1 (“somewhat yes”), 2 (“yes”), 3 (“definitely yes”). We adopted a 6-point Likert scale rather than a binary response scale to increase the sensitivity of our measure. Participants were informed that if they selected any of the “NO” options, all six cards would be displayed again for 2500 ms. If they instead selected any of the “YES” options, six cards would be shown for 500 ms and then 3 of the decoy cards would be removed, thus leaving the outcome card and two additional decoy cards. Participants were informed that if they decided to purchase information this would have a cost between 0 and 3 points. They did not know the exact cost on each trial. This reduces the likelihood that participants would attempt to explicitly calculate the exact instrumental utility of information relative to cost. We did this as we did not want participants to focus their attention and cognitive resources on an exact, explicit, mathematical calculation, to avoid distracting them. Furthermore, in real life the cost of information often cannot be precisely calculated, as in “opportunity costs”. Therefore, we wanted to induce some uncertainty regarding the cost. We tested participants' understanding of the above rules using a questionnaire. Participants who failed this test did not advance on to the lottery task.

After participants decided whether to purchase information, they were asked to rate their current mood by indicating “How happy are you right now” on a 7-point scale (i.e., 0 – “very unhappy”, 1 – “unhappy”, 2 – “somewhat happy”, 3 – “neutral”, 4 – “somewhat happy”, 5 – “happy”, 6 – “very happy”) and to indicate “What do you think the outcome would be?” on a 7-point (i.e., 0 – “certainly loss”, 1 – “loss”, 2 – “likely loss”, 3 – “not sure”, 4 – “likely gain”, 5 – “gain”, 6 – “certainly gain”; Fig. 1). Again, this last query was used to compute subjective uncertainty (**Analysis**).

Subsequently, they were asked whether they wanted to play the lottery. The outcome of the lottery was never revealed. This was done in order to mimic real life scenarios in which we often receive immediate information (e.g., result of a DNA test examining genetic predistortion to cancer) which might be useful for directing future choices (e.g., eating healthier), but the outcome may be revealed years later or sometimes never at all (e.g., whether we end up suffering from cancer). In addition, not revealing outcomes immediately would increase the salience of the information itself. Participants were told that at the end of the task one trial would be randomly selected for the bonus payment. Participants were informed that their bonus payment would be *at least equal* to the outcome on the randomly selected trial (either a gain or a loss if the lottery was played on this trial, or zero if the lottery was passed on that trial) minus the cost of information if it was a trial where information was purchased. Participants were also informed that 33 points were worth approximately £1, and each card could display numbers between −100 and 100. However, all participants received a bonus of £3 upon task completion.

We pre-selected card combinations for each lottery such that to minimize the natural correlations among the task variables: the expected value of the lottery (EV), the standard deviation of the lottery (SD) and the instrumental utility of information (IU; computed by multiplying the objective agency with the difference between averaged EV from all possible information feedback combinations and the EV of the lottery). We generated many card combinations by randomly sampling the cards on each trial. We then chose the ensemble of card combinations which resulted in lowest correlation coefficients between the three factors across lotteries. Such lotteries had the following correlation coefficients between the three factors across lotteries: EV and IU *r* = 0.074 *p* = 0.487, EV and SD *r* = 0.156, *p* = 0.143; IU and SD: *r* = 0.367, *p* = 0.004 (note, that the latter significant medium/low coefficient still allows for the variables to compete for variance in the same model).

In the control studies (*Experiment* 3&*4*), participants played with the same task and 90 lotteries, but they did not provide any subjective ratings. This allowed us to test whether objective measures of instrumental utility, EV and uncertainty were related to information-seeking choices even when participants were not driven to focus on those elements by asking them to introspect about related constructs.

In *Experiment 5*, participants played the same task as in *Experiment 1* and *2*, but their request for information or ignorance was honored on 75% of the trials only. This allowed us to dissociate the effect of receiving information from the effect of selecting it on external and internal outcomes.

To asses participants' attention we added four “catch trials” throughout the experiment. In each catch trial, a question was shown to participants asking whether the card displayed on the screen was a gain (blue) or a loss (red) card. Four catch trials were placed at the 15th, 30th, 45th and 70th trial respectively. Most participants did well on the catch trials as can be observed in the table below (Table 1). Excluding participants that failed >25% of catch trials did not alter the results reported in the main text.

**Table 1:** Participants' attention. In *Experiment 1* none of the participants failed >25% of the catch trials. In *Experiment 2* and *3*, only two participants failed catch trials >25% of the catch trial. In *Experiment 4*, five participants failed >25% of the catch trials. In *Experiment 5*, only one participant failed >25% of the catch trials.

Experiment	Number of Participants who failed >25% catch trials
1	None
2	Two
3	Two
4	Five
5	One

### 2.3 Analysis

#### 2.3.1 Anticipating the impact of information

To examine which factors best account for information purchasing decisions we ran different linear mixed-effects models with information purchasing ratings (from 3 “definitely yes” to −3 “definitely no”) as the dependent variable and the factors described below as fixed and random variables. We also included a random intercept. Our hypothesis was that participants would be more likely to purchase information when they expected information to improve their internal states and external outcomes. Based on our theory of information-seeking motives (Sharot & Sunstein, 2020), we predicted that people would be more likely to purchase information when they expected information to (i) improve their affect; (ii) reduce their uncertainty and (iii) have instrumental value (that is they could use information to direct actions). To test these predictions, we measured the following three factors:

I. *Subjective factors*:

(i) **Anticipated Affect-** Participants' rating of how they expected to feel when information was revealed (from 0 – “very unhappy” to 6 – “very happy”).

(ii) **Subjective Uncertainty**- Participants' subjective uncertainty regarding the outcome (from 0 – “certainly loss” to 6 – “certainly gain”). These were then rescaled to compute participants' subjective uncertainty. In particular, “not sure” was rescaled as 0 (indicating high uncertainty), “likely a loss” and “likely a gain” as (−2), and “certainly gain” or “certainly loss” as (−3) (indicating low uncertainty).

(iii) **Subjective Instrumental Utility** - The subjective instrumental utility of information (sIU) which is equal to the product of the agency the subject believed they had over the gambling decision and the difference in the expected value of the lottery before and after receiving information. It is formally defined as:

(1) $${\mathrm{sIU}}_{t}=\left({\mathrm{aEV}}_{t}-{\mathrm{EV}}_{t}\right)*{\mathrm{Psubjective}}_{t}$$

*Psubjective*_*t*_ is equal to participants' rating of their subjective agency in making the gambling decision. *aEV* is the mean expected value of all possible 3-card combinations of lotteries after receiving information. That is, the mean of all possible combinations of 3 cards of the information feedback (which may or may not include the outcome card). Any time the EV was <0 we assume that the subject will decide to pass (that is not take the gamble) and thus the value is entered as 0. *EV*_*t*_ is the expected value of the lottery before receiving information. Any time the EV was <0 we assume that the subject will decide to pass (that is not take the gamble) and thus the value is entered as 0.

II. *Objective factors*:

i) **Expected value** of the lottery (EV):

(2) $$\;{\mathrm{EV}}_{t}=\sum_{i=1}^{i=6}\left(c_{i}\times p\right)$$

where *c*_*i*_ is the value of the card *i* displayed to participants at trial *t* and *p* is the probability that card *i* will be chosen if the lottery is played and it is equal $\frac{1}{6}$ in every trial t.

ii) **Uncertainty** about the lottery outcome was computed using two alternative methods:

1) As the standard deviation (SD) of the lottery:

(3) $${\mathrm{SD}}_{t}=\sigma_{t}=\sqrt{\frac{\sum_{i=1}^{i=6}{\left(c_{i}-\mu \right)}^{2}}{N}}$$

where *c*_*i*_ is the value of the card *i* displayed to participants at trial *t, μ* is the mean of the lottery and N is the total number of cards in the lottery (i.e., *N* = 6).

2) As entropy (E):

(4) $$\;E_{t}=-\left(p_{\mathrm{win}}*\log\left(p_{\mathrm{win}}\right)+p_{\mathrm{lose}}*\log\left(p_{\mathrm{lose}}\right)\right)$$

where *p*_*win*_ is the probability of win and *p*_*lose*_ is the probability of lose, respectively.

(iii) **Objective Instrumental Utility** (IU) which was computed the same as in the *subjective instrumental utility*, except that the actual probability that the subject will be given the opportunity to make the lottery decision (i.e., objective agency) is entered rather than their subjective perception of that number.

We compared models which include: one, two or three subjective measures; one, two or three objective measures; and a mix of objective and subjective measures. For the objective computation of uncertainty, we included models with either SD or E. We compared these models using the BIC scores. The BIC penalizes for a number of parameters such that a model with more variables does not have an advantage over a model with less variables. A smaller BIC value indicates a better fit. We also compare models using the AIC scores (see **Supplementary Tables**). Again, a smaller AIC value indicates a better fit. As we observed similar results using the AIC and BIC, we include BIC values in the main text and AIC in supplementary material. Additionally, we ran the fitting procedure in both R studio (using nlme package) and in MATLAB (using fitlme function) obtaining identical results (in the **Supplementary Tables** we report the results obtained when fitting the models in MATLAB).

#### 2.3.2 Computing the impact of information

In *Experiment 1* and *2*, we investigated that impact of information over internal (mood and uncertainty reduction) and external (point obtained) states by (i) comparing mood rating on trials when information was purchased vs trials where information was not purchased; (ii) comparing the change between the second and first subjective uncertainty score (calculated as described above) on trials when information was purchased vs trials where information was not purchased; (iii) comparing the average points obtained on trials when information was purchased vs trials where information was not purchased. Points on each trial were computed by subtracting the outcome value from information cost. If information was not purchased then information cost was zero. If the gamble was not played out than the outcome value was zero. Both trials in which participants decided whether to gamble and trials in which the computer made that decision were included.

In *Experiment 5*, we submitted mood rating, the difference in uncertainty reduction (after-before) and proportion of win into three separate ANOVAs with choice (Select Information, Select Ignorance) and outcome (Info Received, Info Withheld) as repeated measures.

## 3 Results

### 3.1 Experiment 1

Participants played a lottery game for 90 trials. On each trial a lottery composed of 6 cards, with each card displaying either a positive or negative value (Fig. 1A) was presented. One of the cards had a star on the back of it, which determined the outcome on that trial if the lottery was played out. Participants were told that on some trials they could decide to play the lottery or pass and on some other trials a computer would randomly decide for them. Prior to the lottery choice, participants were asked to indicate whether they wanted to purchase information about the upcoming lottery on a 6-point Likert scale: −3 (“definitely no”), −2 (“no”), −1 (“somewhat no”), 1 (“somewhat yes”), 2 (“yes”), 3 (“definitely yes”). Participants were informed that if they selected any of the “NO” options, then all six cards were displayed again. If participants selected any of the “YES” options three cards were removed, leaving the outcome card and two additional cards.

To assess participants expectations on how information would impact them, they were asked how they expect to feel if they received information about this gamble on a scale from 0 – “very unhappy” to 6 – “very happy” and what they thought the outcome would be on a scaled from 0 – “certainly loss” to 6 – “certainly gain”. We used the latter rating to compute subjective uncertainty, by rescaling the scores such that the extremes of the scale were scored as high certainty and scores towards the middle of the scale as high uncertainty (see **Methods**). They also indicated their belief of the likelihood that they would be chosen to play the lottery rather than the computer on a scale from 0 – “very unlikely” to 6 – “very likely”.

**Fig. 2:**
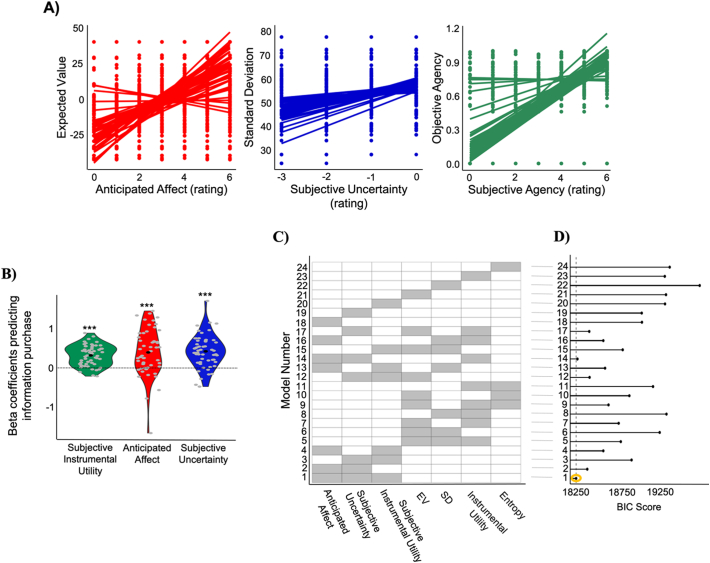
Anticipated impact of information on internal states and external outcomes explains information-seeking. A) The plots show that each subjective measure significantly correlated with the corresponding objective measure: anticipated affect corelates with expected value of the lottery (EV), subjective uncertainty correlates with the standard deviation of the lottery (SD), subjective agency correlates with objective agency. Each line represents one participant. B) Information purchasing decisions were predicted from (i) the subjective instrumental utility of information (which is equal to the product of the subjective agency and the difference in the expected value of the lottery before and after receiving information), (ii) participants' rating of how they expected to feel when information was revealed (anticipated affect) and (iii) participants' subjective uncertainty regarding the lottery outcome. Grey dots represent beta estimates for each participant obtained by fitting the model to each subject individually. C) For each model the corresponding fixed variables included in the model are colored in grey. D) BIC shows that the winning model is the model which includes the three subjective factors and returns the smallest BIC (as shown by the dashed line and yellow circle). * *p* < 0.05, **p < 0.01, ***p < 0.001. (For interpretation of the references to colour in this figure legend, the reader is referred to the web version of this article.)

***Subjective* evaluations of internal and external states explain information-seeking best.** We first examined if participants' subjective assessments were associated with the corresponding objective factors. Indeed, participants' rating of anticipated affect correlated with the expected value of the lottery (EV). The greater the EV the better people expected to feel when information was revealed. This association was shown by correlating EV and expected affect rating across trials for each subject and then comparing the resulting correlation coefficients across participants to zero (mean Pearson *R* = 0.613, SD = 0.258; *p* < 10^−15^; Fig. 2A). Second, participants' ratings of subjective uncertainty correlated both with the standard deviation of the lottery (SD) and entropy. Greater SD and greater entropy were associated with greater perceived uncertainty (correlation with SD: mean Pearson *r* = 0.266, SD = 0.086; *p* < 10^−15^; Fig. 2A; correlation with entropy: Pearson *r* = 0.523, SD = 0.114; *p* < 10^−15^). Third, participants' ratings of subjective agency correlated well with the actual probability that participants would be able to make the gambling choice (i.e., objective agency; mean Pearson *r* = 0.809, SD = 0.259; *p* < 10^−15^; Fig. 2A).

Next, we examined if participants' subjective assessments explained information purchasing decisions. To that end, we ran a linear mixed-effects model with information purchasing ratings (from 3 “definitely yes” to −3 “definitely no”) as the dependent variable and the three subjective factors (anticipated affect, subjective uncertainty, subjective instrumental utility) as fixed and random variables with both random intercepts and slopes. All three variables significantly predicted information purchasing decisions. In particular, participants wanted to purchase information more when they expected it will make them feel better (beta coefficient = 0.411 ± 0.082 (SE), *t* = 5.03, *p* 〈10^−3^), when their subjective uncertainty was greater (beta coefficient = 0.422 ± 0.054 (SE), *t* = 7.86, *p* < 10^−3^), and when the subjective instrumental utility of information was higher (beta coefficient = 0.317 ± 0.034 (SE), *t* = 9.21, *p* < 10^−3^). These results were replicated with a linear regression model fit to each participant individually and then betas were tested across participants against zero (anticipated affect: mean beta = 0.415 t(53) = 5.00, *p* < 10^−5^; subjective uncertainty: mean beta = 0.42 t(53) = 7.5, *p* < 10^−9^; subjective instrumental utility: mean beta = 0.31 t(53) = 8.86, *p* < 10^−11^; Fig. 2B).

A key question was whether participants' subjective estimates better predicted information-seeking choices than objective proxies of these estimates. To address this, we compared the above model (which includes subjective factors) to a model which incorporated the following objective factors: EV as a proxy for anticipated affect; SD or entropy as a proxy of uncertainty and the instrumental utility calculated using objective agency (**Methods**). The ‘subjective model’ fit the data better than the ‘objective model’ as observed by its lower BIC score (Fig. 2C). It also fit the data better than a range of other models where only one or two subjective measures were included; where only one or two objective measures were included; and where a mix of objective and subjective measures were included (see Fig. 2C). Lastly, we tested whether our model that accounts for individual differences was better able to explain the data compared to a model which considers population-level anticipated affect, subjective uncertainty, and subjective instrumental utility. To do so, for each lottery type we computed the average ratings across the population for each of the three factors and entered them in a mixed-model predicting individual information-seeking choices. The “averaged model” did not fit the data as well as our original winning model as evident by the BIC score (*Experiment 1*: BIC averaged model = 18,394; BIC original model = 18,150) suggesting that accounting for individual differences in information-seeking is necessary to better explain the data.

**Fig. 3:**
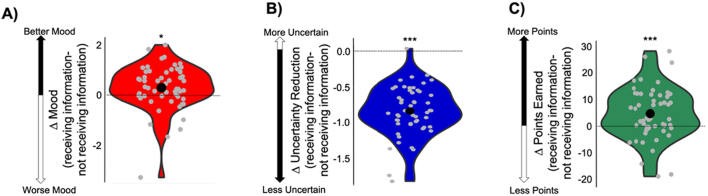
Consequences of Information. Participants were (A) happier, (B) had reduced uncertainty to a greater extend and (C) made better gambling decisions (obtained more points), after receiving information than after remaining in the dark. * p < 0.05, ** *p* < 0.01, *** *p* < 0.001.

**Participants are happier, less uncertain and gain more points when they purchased information relative to when they did not.** We observed that participants sought information more if they *expected* information to positively impact their affect, when they were more uncertain about the lottery outcome and when information could help them gain more points from their gambling decisions (i.e., information had higher instrumental utility). Next, we examined if information consumption, as compared to its avoidance, in fact improved their mood, reduced their uncertainty and led to better gambling decisions. Our results showed that participants were happier when receiving information (M = 2.87, SD = 0.53) relative to when they did not (M = 2.58, SD = 0.74; significant difference between the two: t(53) = 2.43, *p* < 0.05; Fig. 3A), they became less uncertain about the outcome after receiving information (M = − 0.78, SD = 0.30) relative to when they did not (M = − 0.05, SD = 0.21 significant difference between the two: t(53) = − 17.8, *p* < 10^−15^; Fig. 3B); and they made better gambling decisions (gained more points) after receiving information (M = 5.74, SD = 7.34) compared to trials where information was not purchased (M = 0.96, SD = 6.71; t(53) = 3.6, *p* < 10^−3^; Fig. 3C). In *Experiment 5*, we will disentangle the effects of participants' information purchasing *choices* from the effects of receiving information, on the three factors above.

### 3.2 Replication: Experiment 2

The above results were replicated using an independent sample (*N* = 136; based on a power analysis see **Methods**) who completed the same task as in *Experiment 1*.

As in *Experiment 1*, (i) anticipated affect correlated with the EV of the lottery (mean Pearson *r* = 0.54, SD = 0.33; *p* < 10^−15^); (ii) subjective uncertainty correlated with the SD of the lottery, such that when SD was greater people were more uncertain (mean Pearson *r* = 0.27, SD = 0.11; *p* < 10^−15^) and it also correlated with the entropy of the lottery, such that when the entropy was greater people were more uncertain (mean Pearson *r* = 0.541, SD = 0.146; *p* < 10^−15^); (iii) Subjective agency correlated well with the actual probability that participants will be able to make the gambling choice themselves (mean Pearson *r* = 0.79, SD = 0.30; *p* < 10^−15^).

**Fig. 4:**
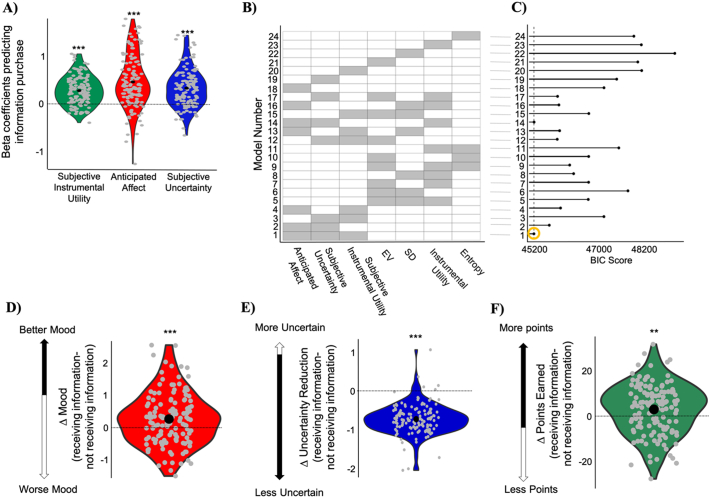
Replication study. A) Information purchasing decisions were predicted from (i) the subjective instrumental utility of information (ii) participants' rating of how they expected to feel when information was revealed (anticipated affect) and (iii) participants' subjective uncertainty. The figure shows individual beta estimates of these predictors for each participant. B) For each model the corresponding fixed variable are presented in grey. C) BIC of the models reveal the winning model is the subjective model which returns the smallest BIC (as shown by the dashed line and yellow circle). Participants were (D) happier, (E) reduced uncertainty to a greater extend and (F) obtained more points after receiving information than after not receiving information. In all the panels, * is *p* < 0.05, ** is p < 0.01, *** is *p* < 0.001. (For interpretation of the references to colour in this figure legend, the reader is referred to the web version of this article.)

We ran the same linear mixed-effects model as in *Experiment 1*, which again revealed that participants wanted to purchase information more when they expected it will make them feel good (beta coefficient = 0.471 ± 0.047 (SE), *t* = 9.98, *p* < 10^−3^), when their uncertainty was greater (beta coefficient = 0.345 ± 0.033 (SE), *t* = 10.3, *p* < 10^−3^), and when subjective instrumental utility of information was higher (beta coefficient = 0.284 ± 0.025 (SE), *t* = 11.19, *p* < 10^−3^). The same results were observed when a linear regression model was fit to each participant separately and then betas tested across participants against zero (anticipated affect: mean beta = 0.47, t(135) = 9.96, *p* < 10^−15^; subjective uncertainty: mean beta = 0.338, t(135) = 10.02, *p* < 10^−15^; subjective instrumental utility: mean beta = 0.286, t(135) = 11.14, *p* < 10^−15^; Fig. 4A). The “subjective model” fit the data better (BIC = 45,203) than the objective model (BIC = 46,697) and all other combination of models (Fig. 4B), including the “averaged model” (BIC averaged model = 45,716) suggesting that accounting for individual differences in information-seeking is necessary to better explain the data.

As in *Experiment 1*, participants were happier when they received information (M = 2.93, SD = 0.56) relative to when they did not (M = 2.66, SD = 0.85; significant difference between the two: t(135) = 3.89, *p* < 10^−3^; Fig. 4C), they became less uncertain after receiving information (M = − 0.77, SD = 0.35) relative to when they did not (M = − 0.05, SD = 0.28; significant difference between the two: t(135) = − 21.34, *p* < 10^−15^; Fig. 4C), and they made better gambling decisions (gained more points) after receiving information (M = 5.37, SD = 7.68) relative to when they did not (M = 2.38, SD = 8.50; significant difference between the two: t(135) = 3.12, *p* < 0.01; Fig. 4C).

The results of this replication study support the findings that participants anticipate the impact of information on internal states and external outcomes and use these subjective evaluations to direct information foraging behavior. As a result, participants improve both internal and external states using information.

### 3.3 Control studies: Experiment 3 & 4

*Experiment 1* and *2* demonstrate that participants are more likely to purchase information when they expect information to induce positive affect, when they are uncertain about the outcome and when information can be used to gain more points. It is possible, though, that participants' tendency to do so was influenced by the fact that their attention was focused on these aspects of the task because we asked them how information would make them feel, how certain they were about the outcome and how likely they were to have agency. We thus conducted a third study on an independent sample (*Experiment 3*) to examine whether participants would make similar decisions even when they were not asked to introspect.

The task was exactly as in *Experiment 1* and *2*, except that participant were only asked to make the information-seeking choices and not asked to provide subjective evaluations. The analysis was exactly as in *Experiment 1* and *2*, however given that there were no subjective measures we only ran the *objective* model. We used the objective model to predict participants' trial-by-trial information choices. The model revealed that participants were more likely to purchase information when EV of the lottery was greater (beta coefficient = 0.346 ± 0.057 (SE), *t* = 6.12, *p* < 10^−3^), when the SD of the lottery was larger (beta coefficient = 0.06 ± 0.023 (SE), *t* = 2.64, *p* = 0.008) and when instrumental utility of information was greater (beta coefficient = 0.596 ± 0.043 (SE), *t* = 14.01, *p* < 10^−3^). The above results were also observed when “entropy” was included in the objective model instead of SD as measure for uncertainty (EV beta coefficient = 0.254 ± 0.06 (SE), *t* = 4.41, *p* < 10^−3^; Entropy beta coefficient = 0.45 ± 0.032 (SE), *t* = 13.86, *p* < 10^−3^; instrumental utility beta coefficient = 0.4 ± 0.034 (SE), *t* = 11.63, *p* < 10^−3^).

**Fig. 5:**
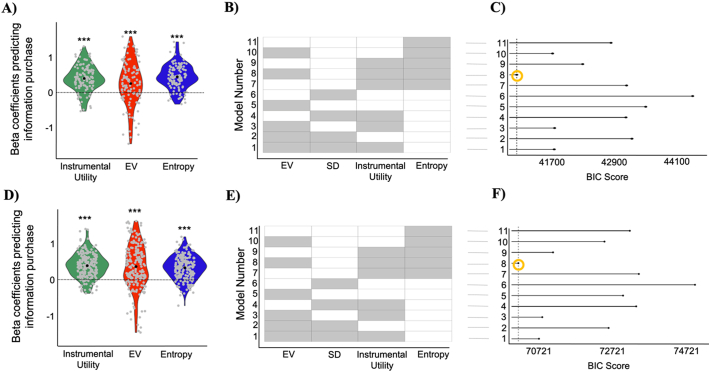
Control study. (A-C) Shows results of *Experiment 3* and (D—F) of *Experiment 4*. *(*A & D) Information purchasing decisions were predicted from (i) the instrumental utility of information, (ii) the lottery EV and (iii) the lottery uncertainty. The figure shows individual beta estimates of these predictors. Beta coefficients of these 3 predictors are all significantly different from zero. (B, E) For each model the corresponding fixed variable included in the model are colored in grey. (C, F) BIC of the model shows the winning model is the objective model with three proxies which returns the smallest BIC (as shown by the dashed line and yellow circle). * *p* < 0.05, ** *p* < 0.01, *** *p* < 0.001. (For interpretation of the references to colour in this figure legend, the reader is referred to the web version of this article.)

The same results were observed when a linear regression model was fit to each participant separately, and the resulting betas were tested across participants against zero with a one-way *t*-test (model1 - EV: mean beta = 0.346, t(118) = 6.09, *p* < 10^−7^; SD: mean beta = 0.062, t(118) = 2.63, *p* = 0.01; instrumental utility: mean beta = 0.596, t(118) = 13.95, *p* < 10 ^−14^; model 2 - EV: mean beta = 0.254, t(118) = 4.39, *p* < 10^−4^; Entropy: mean beta = 0.45, t(118) = 13.8, *p* < 10^−10^; instrumental utility: mean beta = 0.4, t(118) = 11.59, *p* < 10 ^−14^; Fig. 5A). Comparing the two models, the model which includes entropy fit the data best. This model also fit the data better than models with only one or two of these predictors (Fig. 5B).

Similar results were obtained in a replication study (*Experiment 4*). As in *Experiment 3*, participants' information purchase decisions were explained by EV of the lottery (beta coefficient = 0.445 ± 0.043(SE), *t* = 10.29, *p* < 10^−3^), SD (beta coefficient = 0.045 ± 0.021 (SE), *t* = 2.11, *p* = 0.035) and instrumental utility of information (beta coefficient = 0.541 ± 0.028 (SE), *t* = 19.19, *p* < 10^−3^). Similarly, participants' information purchase decisions were explained by EV of the lottery (beta coefficient = 0.376 ± 0.044(SE), *t* = 8.57, *p* < 10^−3^), entropy (beta coefficient = 0.336 ± 0.023 (SE), *t* = 14.67, *p* < 10^−3^) and instrumental utility of information (beta coefficient = 0.394 ± 0.024 (SE), *t* = 16.46, *p* < 10^−3^). The same results were observed when a linear regression model was fit to each individual and then the resulting betas were tested across participants against zero with a one-way t-test (model 1 - EV: mean beta = 0.445, t(199) = 10.26, *p* < 10^−14^; SD: mean beta = 0.045, t(199) = 2.11, *p* = 0.036; instrumental utility: mean beta = 0.541, t(199) = 19.15, *p* < 10 ^−14^; model 2 -EV: mean beta = 0.376, t(199) = 8.55, *p* < 10^−15^; Entropy: mean beta = 0.336, t(199) = 14.65, *p* < 10^−14^; instrumental utility: mean beta = 0.394, t(199) = 16.43, *p* < 10 ^−14^; Fig. 5C). Comparing the two models, the model which includes entropy fit the data best. This model also fit the data better than models with only one or two of these predictors (Fig. 5D).

The results suggest that the tendency to seek information more when it is likely to be “good news” (i.e., when EV of lottery is higher), when uncertainty is high and when it has greater instrumental utility is not a consequence of artificially focusing participants' attention on these factors by asking for their subjective evaluations.

### 3.4 Dissociating information from choice: Experiment 5

In the above experiments we show that people anticipate the impact of information on both internal and external outcomes and use these subjective evaluations to direct information foraging behavior (*Experiment 1* and *2*). In *Experiment 5*, we aimed to dissociate the effect of receiving information from the effect of selecting it on external and internal outcomes. To that end, in *Experiment 5*, participants played the same task as in *Experiment 1* and *2*, but their request for information or ignorance was honored on 75% of the trials only. On 25% of the trials, they received the opposite of what they requested.

First, we tested whether the three subjective factors explained information purchase as in the previous experiments. Indeed, participants wanted to purchase information more when they expected it would make them feel good (beta coefficient = 0.255 ± 0.045 (SE), *t* = 5.73, *p* < 10^−3^), when their subjective uncertainty was greater (beta coefficient = 0.45 ± 0.036 (SE), *t* = 12.46, *p* < 10^−3^), and when subjective instrumental utility of information was higher (beta coefficient = 0.38 ± 0.025 (SE), *t* = 15.16, *p* < 10^−3^). The subjective model with these three factors (BIC = 61,487) fit the data better than all other models except that a model which included anticipated affect, subjective uncertainty and objective IU was equivalent (BIC = 61,486).

Next, we submitted mood rating, difference in uncertainty reduction (after-before) and percent wins into three separate ANOVAs with choice (select Information, select Ignorance) and outcome (information received, information withheld) as repeated measures.

**Mood**. We expected mood to be better when requests were honored relative to when they were not honored. This should generate an interaction between choice and outcome. In particular, on trials when information was received, mood would be better when information was selected than when ignorance was selected. In contrast, on trials when information was withheld, mood would be worse when information was selected than when ignorance was selected.

**Fig. 6:**
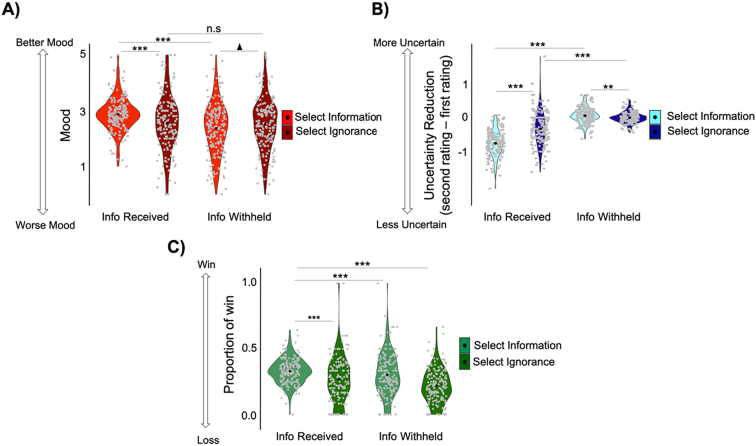
Participants made adaptive information-seeking choices that improved their internal states and external outcomes*.* (A) mood was better and (B) uncertainty was reduced to a greater extent and C) more gambles were won on trials where information was selected and received compared to trials where ignorance was selected but information received (left side of all graphs). * *p* < 0.05, ** *p* < 0.01, *** *p* < 0.001. ▲*p* < 0.1 (trend).

Indeed, we observed an interaction between choice and outcome (F(1,171) = 32.96, *p* < 10^−6^; Fig. 6A). When information was received mood was better when it was desired (M = 2.87, SD = 0.61) than when ignorance was desired (M = 2.55, SD = 1; t(172) = 4.48, *p* < 10^−3^). In contrast, when information was withheld mood tended to be worse when information was desired (M = 2.36, SD = 0.93) than when ignorance was desired (M = 2.51, SD = 0.94; t(174) = −1.87, *p* = 0.063). Moreover, mood was better when participants desired information and received it than when they desired information but did not receive it (t(175) = 7.77, p 〈10^−10^). Together, this pattern of results suggests that participants were making adaptive choices – they selected information on trials where it would increase their mood the most. Importantly, there was also a main effect of outcome, by which mood was better when receiving information than when information was withheld (F(1,171) = 30.27, *p* < 10^−6^) consistent with the idea that information acts as a higher order reward.

**Uncertainty.** For uncertainty reduction (the difference between the second and first uncertainty rating), we also observed an interaction between choice and outcome (F(1,171) = 117.3, p < 10^−15^; Fig. 6**B),** such that uncertainty was reduced more when choices were honored. When information was received uncertainty was reduced more when information was desired (M = −0.757, SD = 0.372) than when ignorance was desired (M = −0.328, SD = 0.501) (t(172) = −10.17, *p* 〈10^−12^). In contrast, when information was withheld, subjective uncertainty was reduced less when information was desired (M = 0.055, SD = 0.22) than when ignorance was desired (M = −0.002, SD = 0.158; t(174) = 2.98, *p* = 0.003).

There was also a main effect of outcome (F(1, 171) = 482.7, p 〈10^−20^), as uncertainty was reduced to a greater extent after information was received than withheld, whether information was desired (t(175) = −27.28, *p* < 10^−15^) or not (t(171) = −8.45, *p* < 10^−14^). There was also a main effect of choice (F(1, 171) = 63.8, p < 10^−10^), driven primarily by the greater reduction of uncertainty when information was received and desired than received but not desired.

**External Outcomes**. As for external outcomes, on trials where subjects decide whether to gamble information is always useful. That is, it can help them make better gambling decisions and thus experience more wins and less losses. On trials where the computer makes the gambling choice, instrumental utility is zero. And in our task, information never has negative instrumental utility. Thus, information should be associated with more wins overall, and indeed it was. Entering proportion of wins into an outcome X choice ANOVA revealed a main effect of outcome F(1,171) = 14.26, p < 10^−3^). Second, because participants were more likely to select information on trials where EV was higher, they should experience more wins and less losses on those trials. This is true regardless of whether they get to make the gambling choice or the computer, and regardless of whether information is received or not. Indeed, participants experienced a greater proportion of wins when they selected information, as observed by a main effect of choice (F(1,176) = 32.72, p < 10^−7^). There was no interaction; we suspect that a task in which information also has negative instrumental utility (not only negative hedonic utility) an interaction would be observed.

Thus, participants experienced most wins when they selected information and received it compared to all other conditions - relative to when they selected ignorance but received information (t (172) = 4.12, *p* = 10^−3^); selected information but it was withheld (t (175) = 2.23, *p* = 0.027); selected ignorance and information was withheld (t (174) = 8.67, p = 10^−10^).

Together, the results suggest that people used information to improve their internal states and external outcomes. When they asked for information and received it, they felt better, their uncertainty was reduced more and they won more, than when they received information without asking for it. While for all three measures there was also a main effect of information (that is mood was better, uncertainty reduced more and wins greater when receiving information), participants requested information when it was likely to improve those outcomes the most.

## 4 Discussion

Here, we show that participants anticipate the impact of information on their internal states and external outcomes and use those predictions to guide information-seeking choices. Participants became happier, less uncertain and made better gambling decisions when they sought information and received it than in all other conditions. These results suggest that people balance considerations of the impact of information on affective, cognitive, and external outcomes when seeking information, to improve all three.

Previous attempts to predict people's information-seeking choices have relied exclusively on objective measures which provide proxies for subjective states (Bromberg-Martin & Hikosaka, 2009; Bromberg-Martin & Hikosaka, 2011; Charpentier et al., 2018; Cogliati Dezza et al., 2017; Cogliati Dezza et al., 2022; Gershman, 2019; Iigaya et al., 2016; Kobayashi et al., 2019; Kobayashi & Hsu, 2019; Wu et al., 2018). For example, it has been shown that seeking information about the outcome of a lottery can be predicted by the standard deviation of the possible outcomes, which is proxy of subjective uncertainty, and expected value of the outcome, which is a proxy of affective reaction (Charpentier et al., 2018). Here, we show that a model which uses subjective evaluations of information's impact on people's affect, uncertainty and external outcomes better accounts for decisions to seek or avoid information than a model which includes only objective proxies of these three factors.

This result is important for explaining individual differences in information-seeking tendencies. That is, while objective measures of these factors in a given situation are the same across individuals, subjective evaluations of these factors may vary. We speculate this is one of the reasons for the large deviations in what people want to know. For example, a recent study (Sunstein, 2019) found that approximately half of individuals surveyed wanted to know if they had a genetic predisposition to cancer, while the other half did not; half wanted to know the estimated global temperature in 2100, half did not; half wanted to know the amount of calories in meal options, half did not. Subjective measures can capture biases and other transformations of objective factors into subjective responses. Another contributor to individual differences in information-seeking choices, is the weight people place on these three factors, that is the importance they place on their affect, uncertainty and external outcomes, when making choices. The balance among these weights might be crucial for determine people's well-being. For example, people who weigh uncertainty more compared to affect may find themselves scrolling through bad news (i.e., doomscrolling) to reduce uncertainty, despite the negatively impact of information on their wellbeing. Note that our study cannot indicate whether the exact weights participants used was optimal in enhancing their well-being.

The current findings provides one of the first empirical evidence, supporting recent information-seeking theories (Golman, Loewenstein, Molnar, & Saccardo, 2021; Sharot & Sunstein, 2020) which suggest that people's decisions to avoid or seek information are jointly influenced by affect, uncertainty and instrumental utility. We are able to do so by using a task in which we independently manipulate uncertainty, instrumental utility, and the likelihood of receiving “good news”. This novel and important feature enabled us to quantify each factor's unique contribution on information-seeking choices. These are often confounded, which is why previous studies have not been able to independently measure the effects of all three factors. We achieved this by varying the probability that the participant was able to use the information to make a decision (agency), which disentangled instrumental utility from expected value and uncertainty.

Our study uses a specific task – a gambling task – to examine information-seeking. Future studies are needed to determine if the findings can be generalized to other contexts (e.g., social domain) and more naturalistic scenarios (e.g., scrolling through social media or browsing information online). Furthermore, other measures could be used to estimate subjective and objective measures. For example, subjective instrumental utility, could be measured by requiring participants to introspect about their likelihood of changing their decisions in response to information. Indeed, one limitation of our study is that the subjective instrumental utility measure contains both participants' interpretation of the likelihood and the unbiased calculation of the effect of information on future choices. Thus subjective instrumental utility and objective instrumental utility are highly correlated. There can be a range of different ways to measure instrumental utility and it is possible that different measures will lead to different results. Moreover, expectations of affective response can be computed by asking subjects not only to predict how they would feel if they received information but also how they would feel if they were to remain ignorant (e.g., Kelly & Sharot, 2021). Finally, other motives may impact information-seeking in other scenarios, such as the need to better comprehend the world around us (Chater & Loewenstein, 2016; Sharot & Sunstein, 2020) or to gain confirmation of our beliefs (Kappes, Harvey, Lohrenz, Montague, & Sharot, 2020).

As massive amounts of information are now easily accessible to people it is important to understand how people decide to seek or avoid information and the impact of these choices on wellbeing. Here, we show that people consider the impact of information on affective, cognitive and external outcomes when deciding to seek or avoid information. People combine these estimates into a calculation of the value of information that can guide information-seeking choices. Failure to do so may be maladaptive. For example, the tendency to “doomscroll” – that is to continuously scroll through bad news despite its negative impact on one's mood - is associated with psychopathology (Price et al., 2022).

## Author contribution

I.C.D. and T.S. conceptualized and designed the experiment. I.C.D. and C. M. carried out the experiment and performed the analyses with guidance from T.S. I.C.D. and T.S. discussed and interpreted the data. I.C.D., C. M. and T.S. wrote the manuscript.

## Declaration of Competing Interest

The authors declare no competing interests.

## Appendix A Supplementary data

Supplementary material Image 1

## References

[bb0005] Bromberg-Martin E.S., Hikosaka O. (2009). Midbrain dopamine neurons signal preference fsor advance information about upcoming rewards. Neuron.

[bb0010] Bromberg-Martin E.S., Hikosaka O. (2011). Lateral habenula neurons signal errors in the prediction of reward information. Nature Neuroscience.

[bb0015] Bromberg-Martin E.S., Monosov I.E. (2020). Neural circuitry of information seeking. Current Opinion in Behavioral Sciences.

[bb0020] Bromberg-Martin E.S., Sharot T. (2020). The value of beliefs. Neuron.

[bb0025] Charpentier C.J., Bromberg-Martin E.S., Sharot T. (2018). Valuation of knowledge and ignorance in mesolimbic reward circuitry. Proceedings of the National Academy of Sciences of the United States of America.

[bb0030] Chater N., Loewenstein G. (2016). The Under-Appreciated Drive for Sense-Making.

[bb0035] Cogliati Dezza I., Cleeremans A., Alexander W. (2022). Independent and interacting value systems for reward and information in the human brain. eLife.

[bb0040] Cogliati Dezza I., Noel X., Cleeremans A., Yu A.J. (2021). Distinct motivations to seek out information in healthy individuals and problem gamblers. Translational Psychiatry.

[bb0045] Cogliati Dezza I., Yu A.J., Cleeremans A., Alexander W. (2017). Learning the value of information and reward over time when solving exploration-exploitation problems. Scientific Reports.

[bb0050] Crupi V., Nelson J.D., Meder B., Cevolani G., Tentori K. (2018). Generalized information theory meets human cognition: Introducing a unified framework to model uncertainty and information search. Cognitive Science.

[bb0055] Friston K. (2010). The free-energy principle: A unified brain theory?. Nature Reviews. Neuroscience.

[bb0060] Gershman S.J. (2019). Uncertainty and exploration. Decision.

[bb0065] Golman R., Gurney N., Loewenstein G. (2020). Information gaps for risk and ambiguity. Psychological Review.

[bb0070] Golman R., Loewenstein G. (2018). Information gaps: A theory of preferences regarding the presence and absence of information. Decision.

[bb0075] Golman R., Loewenstein G., Molnar A., Saccardo S. (2021). The demand for, and avoidance of, information. Management Science.

[bb0080] Gottlieb J., Oudeyer P.Y., Lopes M., Baranes A. (2013). Information-seeking, curiosity, and attention: Computational and neural mechanisms. Trends in Cognitive Sciences.

[bb0085] Hsee C.K., Ruan B. (2016). The Pandora effect: The power and peril of curiosity. Psychological Science.

[bb0090] Iigaya K., Story G.W., Kurth-Nelson Z., Dolan R.J., Dayan P. (2016). The modulation of savouring by prediction error and its effects on choice. Elife.

[bb0095] Kappes A., Harvey A.H., Lohrenz T., Montague P.R., Sharot T. (2020). Confirmation bias in the utilization of others’ opinion strength. Nature Neuroscience.

[bb0100] Karlsson N., Loewenstein G., Seppi D. (2009). The ostrich effect: Selective attention to information. Journal of Risk and Uncertainty.

[bb0105] Kelly C.A., Sharot T. (2021). Individual differences in information-seeking. Nature Communications.

[bb0110] Kidd C., Hayden B.Y. (2015). The psychology and neuroscience of curiosity. Neuron.

[bb0115] Kobayashi K., Hsu M. (2019). Common neural code for reward and information value. Proceedings of the National Academy of Sciences of the United States of America.

[bb0120] Kobayashi K., Ravaioli S., Baranes A., Woodford M., Gottlieb J. (2019). Diverse motives for human curiosity. Nature Human Behaviour.

[bb0125] van Lieshout L.L.F., de Lange F.P., Cools R. (2020). Why so curious? Quantifying mechanisms of information seeking. Current Opinion in Behavioral Sciences.

[bb0130] van Lieshout L.L.F., de Lange F.P., Cools R. (2021). Uncertainty increases curiosity, but decreases happiness. Scientific Reports.

[bb0140] van Lieshout L.L.F., Traast I.J., de Lange F.P., Cools R. (2021). Curiosity or savouring? Information seeking is modulated by both uncertainty and valence. PLoS One.

[bb0145] Oudeyer P.-Y., Lopes M., Kidd C., Gottlieb J. (2016). Curiosity and intrinsic motivation for autonomous machine learning. ERCIM News.

[bb0150] Price M., Legrand A.C., Brier Z.M.F., van Stolk-Cooke K., Peck K., Dodds P.S., Adams Z.W. (2022). Doomscrolling during COVID-19: The negative association between daily social and traditional media consumption and mental health symptoms during the COVID-19 pandemic. Psychol Trauma.

[bb0155] Schulz E., Bhui R., Love B.C., Brier B., Todd M.T., Gershman S.J. (2019). Structured, uncertainty-driven exploration in real-world consumer choice. Proceedings of the National Academy of Sciences of the United States of America.

[bb0160] Schwartenbeck P., Passecker J., Hauser T.U., FitzGerald T.H., Kronbichler M., Friston K.J. (2019). Computational mechanisms of curiosity and goal-directed exploration. Elife.

[bb0165] Sharot T., Sunstein C.R. (2020). How people decide what they want to know. Nature Human Behaviour.

[bb0170] Stigler G. (1961). The economics of information. Journal of Political Economy.

[bb0175] Sunstein C.R. (2019). Ruining popcorn? The welfare effects of information. Journal of Risk and Uncertainty.

[bb0180] Wilson R.C., Geana A., White J.M., Ludvig E.A., Cohen J.D. (2014). Humans use directed and random exploration to solve the explore-exploit dilemma. Journal of Experimental Psychology. General.

[bb0185] Wilson T.D., Gilbert D.T. (2005). Affective forecasting. Current Directions in Psychological Science.

[bb0190] Wu C.M., Schulz E., Speekenbrink M., Nelson J.D., Meder B. (2018). Generalization guides human exploration in vast decision spaces. Nature Human Behaviour.

