# Supplementary material for: People adaptively use information to improve their internal states and external outcomes
Source: Cognition. 2022 Nov;228:105224. doi: 10.1016/j.cognition.2022.105224 (PMC10510028; doi:10.1016/j.cognition.2022.105224)
Supplement: Supplementary file 1 — Supplementary material [file mmc1.docx]

**SUPPLEMENTARY MATERIAL**

Anonymized data and R codes are available at https://github.com/affective-brain-lab/People-adaptively-use-information-to-improve-their-internal-states-and-external-outcomes

**SUPPLEMENTARY TABLES**

**Supplementary Table 1.** AIC estimates for Experiment 1 when fitting the mixed-effects models in R (using nlme package).

| **Model** | **AIC** |
| --- | --- |
| Expected Mood + Subjective Uncertainty + Subjective IU | **18053** |
| Expected Mood + Subjective Uncertainty | 18231 |
| Subjective Uncertainty+ Subjective IU | 18811 |
| Expected Mood+ Subjective IU | 18441 |
| EV + SD + IU | 18637 |
| EV + SD | 19179 |
| EV + IU | 18642 |
| SD + IU | 19268 |
| EV + Subjective Uncertainty + Subjective IU | 18228 |
| Expected Mood + SD + Subjective IU | 18432 |
| Expected Mood + Subjective Uncertainty + IU | 18069 |
| EV + SD + Subjective IU | 18662 |
| Expected Mood + SD + IU | 18409 |
| EV + Subjective Uncertainty + IU | 18225 |
| Expected Mood | 18973 |
| Subjective Uncertainty | 18970 |
| Subjective IU | 19276 |
| EV | 19288 |
| SD | 19729 |
| IU | 19272 |
| EV + Entropy + IU | 18476 |
| EV + Entropy | 18783 |
| Entropy + IU | 19092 |
| Entropy | 19336 |

**Supplementary Table 2.** AIC estimates for the Experiment 2 when fitting the mixed-effects models in R (using nlme package).

| **Model** | **AIC** |
| --- | --- |
| Expected Mood + Subjective Uncertainty + Subjective IU | **45092** |
| Expected Mood + Subjective Uncertainty | 45551 |
| Subjective Uncertainty+ Subjective IU | 45626 |
| Expected Mood+ Subjective IU | 45861 |
| EV + SD + IU | 46586 |
| EV + SD | 47714 |
| EV + IU | 46632 |
| SD + IU | 48058 |
| EV + Subjective Uncertainty + Subjective IU | 48132 |
| Expected Mood + SD + Subjective IU | 45798 |
| Expected Mood + Subjective Uncertainty + IU | 45909 |
| EV + SD + Subjective IU | 46601 |
| Expected Mood + SD + IU | 45778 |
| EV + Subjective Uncertainty + IU | 45742 |
| Expected Mood | 47084 |
| Subjective Uncertainty | 47433 |
| Subjective IU | 48121 |
| EV | 48014 |
| SD | 49030 |
| IU | 49075 |
| EV + Entropy + IU | 48156 |
| EV + Entropy | 46634 |
| Entropy + IU | 46708 |
| Entropy | 47905 |

**Supplementary Table 3.** AIC estimates for the Experiment 3 when fitting the mixed-effects models in R (using nlme package).

| **Model** | **AIC** |
| --- | --- |
| EV + SD + IU | 41630 |
| EV + SD | 43155 |
| EV + IU | 41674 |
| SD + IU | 43040 |
| EV | 43448 |
| SD | 44348 |
| IU | 43081 |
| EV + Entropy + IU | **40913** |
| EV + Entropy | 41636 |
| Entropy + IU | 42213 |
| Entropy | 42783 |

**Supplementary Table 4.** AIC estimates for the Experiment 4 when fitting the mixed- effects models in R (using nlme package).

| **Model** | **AIC** |
| --- | --- |
| EV + SD + IU | 70605 |
| EV + SD | 72523 |
| EV + IU | 70733 |
| SD + IU | 73268 |
| EV | 72945 |
| SD | 74887 |
| IU | 73370 |
| EV + Entropy + IU | **70048** |
| EV + Entropy | 71023 |
| Entropy + IU | 72411 |
| Entropy | 73127 |

**Supplementary Table 5.** AIC estimates for the Experiment 5 when fitting the mixed- effects models in R (using nlme package).

| **Model** | **AIC** |
| --- | --- |
| Expected Mood + Subjective Uncertainty + Subjective IU | **61372** |
| Expected Mood + Subjective Uncertainty | 62170 |
| Subjective Uncertainty+ Subjective IU | 62907 |
| Expected Mood+ Subjective IU | 62710 |
| EV + SD + IU | 62754 |
| EV + SD | 64585 |
| EV + IU | 62796 |
| SD + IU | 64356 |
| EV + Subjective Uncertainty + Subjective IU | 61520 |
| Expected Mood + SD + Subjective IU | 62646 |
| Expected Mood + Subjective Uncertainty + IU | **61371** |
| EV + SD + Subjective IU | 62793 |
| Expected Mood + SD + IU | 62593 |
| EV + Subjective Uncertainty + IU | 61525 |
| Expected Mood | 64911 |
| Subjective Uncertainty | 63587 |
| Subjective IU | 64456 |
| EV | 64991 |
| SD | 65982 |
| IU | 64425 |

**Supplementary Table 6.** AIC and BIC estimates for the Experiment 1 when fitting the mixed-effects models in MATLAB (using fitlme function).

| **Model** | **BIC** | **AIC** |
| --- | --- | --- |
| Expected Mood + Subjective Uncertainty + Subjective IU | **18150** | **18053** |
| Expected Mood + Subjective Uncertainty | 18296 | 18231 |
| Subjective Uncertainty+ Subjective IU | 18876 | 18811 |
| Expected Mood+ Subjective IU | 18506 | 18441 |
| EV + SD + IU | 18735 | 18637 |
| EV + SD | 19244 | 19179 |
| EV + IU | 18707 | 18642 |
| SD + IU | 19333 | 19268 |
| EV + Subjective Uncertainty + Subjective IU | 18326 | 18228 |
| Expected Mood + SD + Subjective IU | 18529 | 18432 |
| Expected Mood + Subjective Uncertainty + IU | 18167 | 18069 |
| EV + SD + Subjective IU | 18760 | 18662 |
| Expected Mood + SD + IU | 18506 | 18409 |
| EV + Subjective Uncertainty + IU | 18322 | 18225 |
| Expected Mood | 19012 | 18973 |
| Subjective Uncertainty | 19009 | 18970 |
| Subjective IU | 19314 | 19276 |
| EV | 19327 | 19288 |
| SD | 19768 | 19729 |
| IU | 19311 | 19272 |
| EV + Entropy + IU | 18574 | 18476 |
| EV + Entropy | 18847 | 18783 |
| Entropy + IU | 19156 | 19092 |
| Entropy | 19375 | 19336 |

**Supplementary Table 6.** AIC and BIC estimates for the Experiment 2 when fitting the mixed-models in MATLAB (using fitlme function).

| **Model** | **BIC** | **AIC** |
| --- | --- | --- |
| Expected Mood + Subjective Uncertainty + Subjective IU | **45203** | **45092** |
| Expected Mood + Subjective Uncertainty | 45626 | 45551 |
| Subjective Uncertainty+ Subjective IU | 47127 | 45626 |
| Expected Mood+ Subjective IU | 45935 | 45861 |
| EV + SD + IU | 46697 | 46586 |
| EV + SD | 47788 | 47714 |
| EV + IU | 46706 | 46632 |
| SD + IU | 48132 | 48058 |
| EV + Subjective Uncertainty + Subjective IU | 48132 | 48132 |
| Expected Mood + SD + Subjective IU | 45909 | 45798 |
| Expected Mood + Subjective Uncertainty + IU | 45208 | 45909 |
| EV + SD + Subjective IU | 46712 | 46601 |
| Expected Mood + SD + IU | 45890 | 45778 |
| EV + Subjective Uncertainty + IU | 45853 | 45742 |
| Expected Mood | 47129 | 47084 |
| Subjective Uncertainty | 47478 | 47433 |
| Subjective IU | 48165 | 48121 |
| EV | 48059 | 48014 |
| SD | 49075 | 49030 |
| IU | 48156 | 49075 |
| EV + Entropy + IU | 46186 | 48156 |
| EV + Entropy | 46708 | 46634 |
| Entropy + IU | 47537 | 46708 |
| Entropy | 47950 | 47905 |

**Supplementary Table 7.** AIC and BIC estimates for the Experiment 3 when fitting the mixed-models in MATLAB (using fitlme function).

| **Model** | **BIC** | **AIC** |
| --- | --- | --- |
| EV + SD + IU | 41740 | 41630 |
| EV + SD | 43228 | 43155 |
| EV + IU | 41747 | 41674 |
| SD + IU | 43113 | 43040 |
| EV | 43492 | 43448 |
| SD | 44391 | 44348 |
| IU | 43125 | 43081 |
| EV + Entropy + IU | **41022** | **40913** |
| EV + Entropy | 41708 | 41636 |
| Entropy + IU | 42285 | 42213 |
| Entropy | 42827 | 42783 |

**Supplementary Table 8.** AIC and BIC estimates for the Experiment 4 when fitting the mixed-models in MATLAB (using fitlme function).

| **Model** | **BIC** | **AIC** |
| --- | --- | --- |
| EV + SD + IU | 70721 | 70605 |
| EV + SD | 72601 | 72523 |
| EV + IU | 70810 | 70733 |
| SD + IU | 73346 | 73268 |
| EV | 72991 | 72945 |
| SD | 74933 | 74887 |
| IU | 73417 | 73370 |
| EV + Entropy + IU | **70165** | **70048** |
| EV + Entropy | 71101 | 71023 |
| Entropy + IU | 72489 | 72411 |
| Entropy | 73174 | 73127 |

**Supplementary Table 9.** AIC and BIC estimates for the Experiment 5 when fitting the mixed-models in MATLAB(using fitlme function).

| **Model** | **BIC** | **AIC** |
| --- | --- | --- |
| Expected Mood + Subjective Uncertainty + Subjective IU | **61487** | **61372** |
| Expected Mood + Subjective Uncertainty | 62247 | 62170 |
| Subjective Uncertainty+ Subjective IU | 62983 | 62907 |
| Expected Mood+ Subjective IU | 62786 | 62710 |
| EV + SD + IU | 62869 | 62754 |
| EV + SD | 64662 | 64585 |
| EV + IU | 62873 | 62796 |
| SD + IU | 64433 | 64356 |
| EV + Subjective Uncertainty + Subjective IU | 61635 | 61520 |
| Expected Mood + SD + Subjective IU | 62761 | 62646 |
| Expected Mood + Subjective Uncertainty + IU | **61486** | **61371** |
| EV + SD + Subjective IU | 62908 | 62793 |
| Expected Mood + SD + IU | 62708 | 62593 |
| EV + Subjective Uncertainty + IU | 61640 | 61525 |
| Expected Mood | 64957 | 64911 |
| Subjective Uncertainty | 63633 | 63587 |
| Subjective IU | 64502 | 64456 |
| EV | 65038 | 64991 |
| SD | 66028 | 65982 |
| IU | 64471 | 64425 |
